# Why people engage in a weight loss intervention at their workplace - a stratified case study

**DOI:** 10.1186/s12889-018-6346-0

**Published:** 2019-01-07

**Authors:** Jeanette Reffstrup Christensen, Majda Pajevic, Pia Maria Ilvig, Karen Søgaard, Christina Jessen-Winge

**Affiliations:** 1 Department of Public Health, University of Southern Denmark, JB Winsløwsvej 9A, 5000 Odense C, Denmark; 2 Department of Sports Science and Clinical Biomechanics, University of Southern Denmark, Campusvej 55, 5230 Odense M, Denmark; 3 Department of Clinical Research, University of Southern Denmark, Winsløwparken 19, 5000 Odense C, Denmark; 4 Department of Occupational Therapy, Institute of Physiotherapy and Occupational therapy, Metropolitan University College, Sigurdsgade 26, 2200 Copenhagen N, Denmark

**Keywords:** Health-promotion program, Lifestyle engagement, Physical detoriation, Health-care workers, Health personnel, Municipalities, Joint responsibility, Qualitative interviews

## Abstract

**Background:**

The prevalence of obesity has increased significantly worldwide within the last decade. As obesity is recognised as a contributing factor when developing various health threatening chronic diseases, prevention initiatives focusing on weight loss are becoming more important. Because of the time spent at the workplace, workplaces can be optimal arenas for weight loss programs and these programs have been effective to decrease body weight. Thus, reasons for engaging in weight loss interventions needs exploring, in order to engage more workplaces in weight loss interventions. Such information provides important knowledge that may help to inform decisions of municipalities, employers and other public health decision makers, when and if implementing weight loss interventions. The aim of this study was therefore to explore reasons for employee engagement in weight loss projects at the workplace and the incentives a municipality, a manager at a home-care centre, and a project manager have to launch such project.

**Methods:**

A stratified case study was conducted. A representative from the municipality, the manager at a home-care centre, the project manager of the weight loss intervention and six health-care workers were interviewed at the end of a one-year weight loss intervention at the workplace. Data were analysed using Systematic Text Condensation.

**Results:**

Analysis identified different views and considerations for engaging in a weight loss intervention at the workplace. For the representative of the municipality the possible economical gain of the project was in focus. The project manager and the manager of the home-care centre both reflected mainly on improvement of the healthcare workers health. For the project manager, achieving good scientific results was highlighted as well. However, the employees were influenced by several factors, such as their own health and weight loss, the pressure from the environment and their struggle for recognition.

**Conclusions:**

This study concluded that if targeting the increasing worldwide obesity problem through workplace initiated weight loss programs, the sales pitch to managements and employers have to be tailored in order to increase the participation and the motivation for the initiative.

**Trial registration:**

ClinicalTrial.gov: NCT01015716, registration data 14.12.2010 (Prospectively registered).

## Background

Obesity is recognised as a contributing factor when developing various diseases, including hypertension, diabetes, heart diseases, dyslipidaemia, cancer and other chronic diseases [1]. Obesity can also increase the risk of musculoskeletal pain, cost in medical care, lost productivity and income, sickness absence and early retirement [1–3]. The prevalence of obesity has increased significantly worldwide within the last decades [4]. In Denmark it is estimated that 1400 people die due to obesity every year, thus making obesity a severe health problem [3]. Therefore, health-promotion and prevention initiatives focusing on weight loss are becoming more important, both at an individual and at a society level.

Many governments worldwide are responsible for conveying information to their populations about health-related risk factors and how to prevent an unhealthy lifestyle. In 2012–2013, the Danish Health Authority developed health-promotion packages, covering focus areas such as increasing physical activity, providing knowledge about healthy food and advices on prevention of obesity [5]. The aim was to support health promotion in the municipalities and to ensure that the local communities could provide a healthy environment as well as health-promoting and disease-preventing activities and facilities [5]. Reducing morbidity and mortality related to obesity is thus considered to be a common concern, and the responsibility is to be shared among the individual, their family, local networks, the local communities, the municipalities, the regions and the state [5, 6].

Health-promotion programs related to workplaces are effective in decreasing body weight [7, 8]. Because of the amount of time spent at the workplace, workplaces can be an optimal setting for weight loss programs [9]. A Danish study - “Frame for interventions for preserved work ability, long term effect” - among health care workers (HCW’s), took place at two home-care centres in Jutland (FINALE-Health). The study showed a positive effect in reducing body weight by 6 kg, Body Mass Index (BMI) by 2.3 and body fat percentage by 2.8 within a 12 months intervention period [10]. The FINALE-Health study, along with other weight loss studies, shows the effects of a weight loss intervention, but do not explore reasons for engaging in weight loss interventions. Based on interviews with informants who had different roles in the FINALE-Health project this study provides a unique perspective to why a municipality, management, project manager and HCW’s participated in a health promoting and weight loss intervention. Such information provides important knowledge that may help to inform decisions of municipalities, employers and other public health decision makers, when and if implementing weight loss interventions.

### Aim

The aim was to explore reasons for HCW’s engagement in weight loss projects at their workplace and the incentives a municipality, a manager at a home-care centre, and a project manager have to launch such projects.

## Methods

### The FINALE-health study

Three Danish municipalities in Jutland were contacted by email and invited to participate in the FINALE-Health project. Randers municipality immediately agreed to participate. The two remaining municipalities were then given written information that the project had the participants needed for the study, and the two remaining invitations were redrawn. Randers municipality had nine care areas, and two care areas were drawn to participate. The same manager led both home-care centres. The home-care centres showed great interest in increasing their employers’ health, had a suitable amount of HCW’s and were not already involved in other health-promotion projects. For detailed description of workplace recruitment see Christensen at al. 2011 [1]. The intervention lasted for 12 months with 144 employees participating, divided in a control and an intervention group. The study was designed as a singled-blinded RCT. Tests were performed at baseline and after three and 12 months. The intervention combined nutrition counselling, cognitive behavioural training and targeted physical exercise, and aimed to increase weight loss and work capacity among HCW’s [10].

### Design and participants

A stratified case study was conducted to explore the informants’ reasons for participating in the weight loss project. The informants were selected among those who in different roles took part of the project; a representative from the municipality, the manager at the home-care centre, the project manager and six healthcare workers, summing nine informants. Among the HCW’s, two informants had participated in the intervention, two had been in the control group and two had not been part of the project. The HCW’s are presented with different age, weight, weight loss and job (evening and day shifts). This variation in involvement and characteristics was prioritized to cover a broad spectrum of potential issues for participation.

### Data collection and analysis

Data was collected within a months after the 12 months intervention period using individually semi-structured qualitative interviews. An interview guide was developed using observations from the FINALE-Health study, review of the literature and informal talks with participants and managers (Table 1). The questions in the interview guide were open-ended and follow-up questions were asked if clarification was needed. All the interviews were conducted face-to-face at the home-care centre in an undisturbed setting by the same researcher (KS). The duration of the interviews varied from 20 to 60 min and was all audio-recorded. The interviews were transcribed verbatim and analysed using Malterud’s systematic text condensation, which is a descriptive and explorative step-by-step method for analysis of qualitative data [11]. The method is inspired by phenomenology and presents the experiences of the informants in their own words. The procedure consists of the following steps: 1) Overall impression and identification of themes; 2) identification and sorting of meaning units, developing from themes to codes; 3) condensation, going from code to meaning; 4) synthesizing of condensation, development of descriptions and concepts. Analysis was data-driven and quotations from participants were used to support the study findings [11]. KS performed analysis in cooperation with JRC. The interviews were conducted in Danish; however, a professional translator translated the quotations used in the present paper from Danish to English. Informed consent was obtained from all informants and they were assured anonymity and confidentiality. All interviews began with a briefing and ended with a de-briefing, where the informants had the opportunity to ask questions if needed.

**Table 1:** Interview guide

Interview questions	
Motivation	
- Can you tell me, what made you participate in the project?	
- Why would you like to lose weight / change habits?	
- Why do you think there are some who have chosen not to join the project?	
- What role do you think the workplace should play in relation to employee health?	
- Why is health / slenderness important? (goal or means)	
Personal effect / importance of participation	
- How has it been to participate in the project?	
- Can you tell what you think you’ve got out of the project?	
- What have you learned? And was it something you didn’t know that already?	
- How have your habits changed? (has something happened?)	
- Why is it a good thing that you have started to exercise more/eat healthier?	
- Can you explain how the project has affected you mentally (mood)?	
- Why did not you lose weight before?	
Workplace – norms	
- How do you think the employees eat here in general? (Healthy/unhealthy)	
- Has it changed since the project started?	
- How would you rate the employees’ overall physical activity level?	
- Has it changed?	
- Does other peoples’ focus on health matter to you and your habits?	
- Do you eat more healthy / unhealthy if others do too?	
- Are you affected by how much exercise others at your workplace do?	
- Can you explain what that means, what others think of your efforts (eat healthy and exercise)?	
Community	
- How has the project affected the workplace in addition to influencing diet and exercise?	
- Has it meant something that some of your collegues had to go from the work to participate?	
- Are there any people, other than yourself, who have had an impact on your lifestyle change?	
- How has it been to exercise and participate in the project with your collegues?	
- How has it been to talk about diet and problem solving with your collegues?	
- How do you think it would have been different, if you had not been in a team, but should participate alone?	
- What had it meant, if a friend or family member could have participated as well?	

## Results

The analysis is structured in three main themes; *1) municipality and management considerations, 2) project manager considerations, and 3) employee considerations and positions*. The first two themes are analysed based on the informants from the municipality and the management, while the third main theme are identified from the HCWs and three subthemes were identified: *a. focus on health and weight loss, b. pressure from the environment* and *c. struggle for recognition.*

### Municipality and management considerations

The municipality and the management at the home-care centre both were motivated by a possible economical gain of the project, however the manager also cared for the health of her employees. For the municipality, the primary reason for conducting the intervention was to provide a good service for the elderly and secondary to promote health of the employees.

“I have to deliver a product. The fact that my employers need to be healthy is not the main product, even though I would like it to be. The main product is that they (the HCW’s) are there for the elderly. I believed that the HCW’s possibly could have fewer sick days and remain longer on the job market than they otherwise would have.” (Representative of the municipality)

For the manager at the home-care centre the initial thoughts were that the project was well suited with existing initiatives, but the manager also wanted to participate for the care of her employees and because the project provided knowledge on healthy living.

“I hoped of course to get some employees who have gained insight in how their bodies function physically and how their body feels when it’s fit. Also in relation to diet: - what is a healthy regular diet, without being fanatical. You could say that everyone gets a picture of what a healthy lifestyle is. And I was also hoping that it could provide some unity […] and I was hoping, of course that it could provide something regarding absence due to sickness.” (Manager at the home-care centre)

All the informants that were involved in the weight loss intervention had considerations regarding the health of the HCW’s, but for the manager the primary goal was to inform and educate her employees on healthy living. When asked if the home-care centre had an obligation to provide health-promoting initiatives, the manager replied:

“I don’t think that we are obliged, but I think that you as an employer, as a municipality, as a human being have a sort of social task or duty to help each other to live healthy, without it being annoying, boring or tiresome.” (Manager at the home-care centre)

### Project manager considerations

The project manager had a scientific approach in her motivation for participating in the weight loss project. The project manager was mainly concerned with helping a group of people whom she believed struggled to optimize their health on their own. When asked what she wished to achieve with the project and what the project could change, she replied:

“…to ensure that they (the HCW’s) are not sick, make sure that they do not get musculoskeletal pain. Yes, to avoid sickness absence and to prevent the HCW’s from being worn out and to avoid them being injured in the long run. That was the overall purpose of the project.” (Project manager)

Her focus was primarily on the physical and mental improvement that the project could provide for the participants.

“Evidence-wise, we know that the more the HCW’s participate in such projects, the higher the probability that the projects have a positive effect and therefore I obviously wanted as many people (HCW’s) as possible to participate in the project.” (Project manager)

Hereby, the project manager puts much emphasis on what provides the best results. Her concerns are firstly scientifically and secondly how to increase the well-being of the HCW’s.

Therefore, it can be acceptable if some feel pressured to participate in the project, if it can help them improve their health.

“There are some who may feel pressured to participate. Now we know that there is a correlation between obesity and constantly having back pain. Especially in this line of work (health-care) because the HCW’s need to pull and push and be close to the burden (the elderly) that they are moving. If you are overweight, or especially if you are a person with obesity, you can’t (the HCW) come close to the elderly, and your reaching distance will be very long. This increases the pressure on the spine and joints, increasing the probability for injury and musculoskeletal pain. In the retirement rates within the HCW’s we can also see that many can’t manage this job in a lifespan. So knowing this, I think we have a co-responsibility - or should we just close our eyes? So the question is whether it is okay that some feel pressured to do something. But of course there will be some who feel pressured to join, even though they do not want to.” (Project manager)

The project manager express the opinion that we as a society have an obligation to help the employees at risk of wearing themself out, even if they feel a bit pressured to participate. With this standpoint the project manager shares the ideas of joint responsibility presented by the government and the manager at the home-care centre.

### Employee considerations and positions

The employees were influenced by several factors when engaging in a weight loss project. These can be classified as *a. focus on health and weight loss, b. pressure from the environment and c. struggle for recognition.*

#### Focusing on health and weight loss

One informant’s motivation for involvement in the project was to improve her health. She stated a desire to lose weight and hoped that the project would give her the necessary help to succeed. When realising her body-age measured at baseline, it was no longer a question of whether she should lose weight, but a feeling of wanting to do better.

“I thought: ‘ that is too bad, it’s too embarrassing, you can do better’. I didn’t think I was that old, because I feel fresh and there was nothing (wrong). I did not think I had difficulty doing anything. I thought that everything was easy and I could ride the bicycle and I could [hesitation] no, I could not run, but I did think I could walk fast. It was not as if I was troubled by my weight.” (Informant 1)

Another participant also wished to lose weight and maintain the weight loss. She clarifies:

“…I know very well that you should lose weight, and I know very well what to do to prevent all those lifestyle diseases. I am in the public health sector, I know very well what I should eat and not eat and I have to advise others, so it does not help that one becomes heavier and heavier and then you tell Mrs Petersen: ‘you jolly well need to eat healthy and then you look like a bear. It doesn’t make sense’.” (Informant 2)

Being an individual with obesity and advising others does not coincide. In order to do her job properly and advice the elderly to a healthy diet, the informant feels that she must lose weight.

#### Pressure from the environment

For some participants there could have been a pressure to participate in the project. One of the informants, not only felt a pressure from the workplace but also from the project manager. She felt that participation was mandatory.

“It was spruced up completely, it was a bit to much. I know, at least in our workgroup, we would perhaps like to do it in our own pace. We thought it was a little too much. So you get a bit, not antipathy, but you are still a little like: ‘they shouldn’t be the ones to decide if I should participate or not. It was a bit like that’.” (Informant 3)

The feeling of owing the workplace to do something about your health can put even more pressure on the HCW’s, although no one expressed that it was the case.

“I am certainly proud to tell where I work. Everyone is very envious of the project and say: ‘Wow, that’s amazing, how can it be done?’ And then I say: ‘That is because they want us to be healthy’, then I think we owe it to them to do something about it.” (Informant 1)

#### Struggle for recognition

Other people’s views and meaning seems to have played a role in the HCW’s decision to participate in the project.

“Well simply because, I’d probably like to be healthy, I want to be flawless and you are not flawless, when you are fat and chubby […] my doctor […] when I had visited to get weighed. He said: ‘You are too smart to look like you do.’ And it’s a bit true, because you know it well. It’s just to get started.” (Informant 1)

She clearly agrees with her doctor that it is not wise to be an individual with obesity. The doctors’ statement illustrates the importance of looking healthy and how we perceive other people. The way we are perceived can be connected with a desire to be accepted and be part of a unity. As one informant elaborated on why she changed her mind in participating in the project, she said:

“Just to be part of that community they had. At the beginning it was because you actually felt a bit left out: then they went biking and then they did something else. So you felt a bit left out.” (Informant 4)

This informant was influenced by her surroundings and the values at the workplace. By joining the values at the workplace, one can align and gain recognition from other colleagues. But it was not only at the workplace it mattered what people thought.

“When I run and my neighbour says, ‘ Well - are you off again? I’ll say it is the third time this week’. It is just completely different from going for a walk as I did before.” (Informant 5)

“I didn’t care if I weighted 88 kilos as I do today. That is not the most important thing, because people cannot se that I weight that much, but it is important that people can see: ‘Oh my, you have lost some weight’. Yes I have!” (Informant 2)

Just like informant 5, informant 2 shared the longing for recognition from other people. By eating healthy and exercising, the HCW’s can gain recognition from their surroundings.

## Discussion

The aim of this study was to explore, why people engage in weight loss projects at their workplace and why such projects are initiated. It became clear that the participants had different reasons for participating. For the representative in the municipality, focus was on the possible economical gain of the project. The project manager and the manager at the home-care centre reflected mainly on the HCW’s health improvements and on achieving valid scientific results. For the employees, loosing weight and improving health was important, but also the pressure from the environment and a struggle for recognition were important factors.

### Productivity and economics

The fact that the municipality was focused on a possible economical gain does not come as a surprise, as obesity is a recognized cost to the workplace as well as to the society. That the management wished to engage in weight reduction to reduce sickness absence and increase work productivity, in order to provide a good service, is supported by other findings. In a study by Gates, employees with a high BMI were significantly less productive than employees with a low BMI [12]. Another study found that workers with obesity were more likely to report lost productivity time, compared to normal or overweight workers [13]. Goetzel et al. also found that employees with obesity are more expensive due to sickness absence, productivity losses and medical care, compared to employees with normal weight [14]. Results from a simulation model on workplace obesity interventions showed that a weight loss of at least 5%, could result in annual savings for medical care and reduction in sickness absence [15].

### Health improvements

The primary goal for both the project manager and the manager at the home-care centre was to improve the health of the HCWs. They agreed with the government approach of a healthy lifestyle being a shared responsibility. The managers expressed desire to help promoting health of their employees is described as a will or need to take care of a specific group, recognized as being in need of such attention. However, as a general tendency in the society, this urge to help a specific group is not unproblematic and at the workplace it may change the relations between employer and employees [16]. The care is usually well intentioned and is often perceived as positive by the recipient, but the outcome is not necessarily positive. Not because the help is not beneficial, but because it simultaneously can leave the recipients powerless. By caring for others, the provider is emphasizing that the recipients are unable to take care of themselves. This can leave the recipients powerless and give more power to the provider. On the other hand, some studies show that participants who enter a weight loss project have a need for additional support. By providing knowledge on weight loss and a healthier lifestyle, these projects in fact helped the participants in obtaining a weight loss [17]. Thus, the project manager and the manager at the home-care centre’s desire to improve the HCW’s health, can both be seen as leaving the HCWs powerless, but also as means to empower the HCWs in order to achieve a healthier lifestyle.

### Social inclusion and recognition

Most of the employee’s were motivated to participate because of a desire to live healthier and to lose weight. This is consistent with findings by Jain et al. and Herriot et al. [3, 18]. They describe a desire to improve appearance and increase fitness. Although, the findings in this study is in accordance with other studies, not all participants in weight loss interventions prioritise their own health, despite known risks and prevention-strategies, as shown in a study by Morrison et al. [19].

The informant’s describe a feeling of pressure to participate from their environment and this is corroborated by findings in a study by Whale [20]. The latter describe that the participants felt a social pressure to lose weight because the government and the media, portrayed a powerful positive image of thinness and beauty [20]. On the other hand there can be a pressure from the HCWs own social relations and interaction with the obesogenic environment, undermining a weight loss [17, 20]. This is a contradiction to the environmental pressure identified in the present study.

The HCW’s experience of pressure to participate in the weight loss intervention may also be interpreted as a desire for social inclusion and a struggle for recognition. Being obese is often seen as a personal and a moral failure, and those who do not comply with the social expectations of being a responsible and a healthy citizen often experience stigmatization, social rejection and exclusion [21]. Other peoples and our own wishes, expectations and actions, influence a desire to gain recognition and to try to live up to the norms in a society, in order to be included in the community [22–24]. It is important to signal that you can manage yourself and live up to the norms of accepted appearance, as obesity is seen as a sign of weakness [25]. This pressure may be especially hard for HCWs who feel that they should be role models as workers in the health care sector. The need to be a role model is consistent with findings by Puhl et al. from 2013. The authors examined the effect of physicians’ body weight on their patients attitudes and concluded that health providers’ excess body weight may negatively affect their patients’ perception of their credibility and their level of trust and that their patients were less likely to follow their advice [26]. Based on these considerations, it is understandable if HCWs with obesity feel compelled to lose weight. The help they can get from the FINALE-Health can therefore seem appealing, and a desire for social inclusion rather than an internal motivation for losing weight. It should therefore be a concern in future weight loss interventions, how to best motivate employers such as health care workers. They are characterised as people in the lower end of the social classes, who are known to have a higher frequency of obesity problems, as well as risks for lifestyle diseases. We must therefore strive to find internal motivation factors that can help lover social class groups to succeed in obtaining a healthy lifestyle, as well as helping with external motivation factors, to overcome equality in health.

The present study is nested within the Finale-Health study [1]. It aimed primarily at decreasing musculoskeletal pain by increasing general health. The intervention was therefore targeted to increase physical healthy everyday activities, physical fitness and muscle strength, and decrease blood pressure, waist circumference, body fat and BMI. The study significantly improved all these health measurements besides muscle strength and musculoskeletal pain after a one-year follow-up [10], and was thus improving general health among health-care workers. As health-care workers are a high-risk population of several lifestyle diseases, it is important to find ways to improve their health. Several studies underpin the difficulties with maintaining a sustainable weight loss after a weight loss intervention. As general health can be improved and risk of lifestyle diseases decreased by increasing physical healthy activities and a healthier diet, future studies, also at the workplaces, should maybe focus at these improvements, especially if the targeted population do not have internal motivations to lose weight [27, 28].

### Methodological reflections

To obtain the broadest perspective of the subject, the researchers choose the principle of maximum variation in the selection of the informants. This gave the researchers a wider range of knowledge and the opportunity to select informants who could provide a large spectrum of information. Despite the effort, it is not a guarantee, that all views are represented. More views could have been obtained, had we used a larger sample and gathered data using another approach, such as open questionnaires. On the other hand this would hinder the possibility to ask further questions in depth, as we wished for in the present paper.

## Conclusion

This study concluded that if targeting the increasing worldwide obesity problem through workplace initiated weight loss programs, the sales pitch to managements and employers have to be tailored in order to increase the participation and motivation for the initiative.

## Abbreviations

BMI: Body Mass Index
FINALE-Health: Frame for InterveNtions for preserved work Ability, Long term Effect” - among health care workers
HCW: Health Care Worker
